# VRE in cirrhotic patients

**DOI:** 10.1186/s12879-019-4352-1

**Published:** 2019-08-13

**Authors:** Melissa Barger, Emily Blodget, Sol Pena, Wendy Mack, Tse-Ling Fong

**Affiliations:** 1 Ventura County Medical Center, 300 Hillmont Ave., Ventura, CA 93003 USA; 2 Division of Infectious Diseases, University of Southern California Keck School of Medicine, 2020 Zonal Ave. IRD Room 436, Los Angeles, CA 90033 USA; 3 Kaiser Permanente of Southern California, 9333 Imperial Highway, Downey, CA 90242 USA; 4 University of Southern California Keck School of Medicine, SSB 202Y 2001 N. Soto Street, Los Angeles, CA 90033 USA; 5 Division of Gastroenterology, University of Southern California Keck School of Medicine, 1520 San Pablo Suite 1000, Los Angeles, CA 90033 USA

**Keywords:** Vancomycin-resistant enterococcus, Cirrhosis, Liver disease

## Abstract

**Background:**

Vancomycin resistant enterococci (VRE) infections are of increasing concern in many hospitalized patients. Patients with cirrhosis are at added risk of infection with VRE, with associated increased risk for complications from infections. The goals of this study were to: [1] identify risk factors for VRE amongst cirrhotic patients before liver transplantation, and [2] evaluate risk of morbidity and mortality at 30-days and one-year after VRE infection.

**Methods:**

Chart review of 533 cirrhotic patients hospitalized at a tertiary medical center was performed. Patients infected with VRE (*n* = 65) were separately compared to patients infected with gram-negative organisms (*n* = 80) and uninfected patients (*n* = 306).

**Results:**

In multivariable logistic regression analyses, female gender (OR 3.73(95% CI1.64,8.49)), severity of liver disease measured by higher Child Pugh scores (OR 0.37(95%CI 0.16,0.84)), presence of ascites (OR 9.43(95% CI 3.22,27.65) and any type of dialysis (OR 3.31,95% CI (1.21,9.04), oral antibiotic prophylaxis for spontaneous bacterial peritonitis and rifaximin use were statistically significantly associated with VRE infection (OR 2.37 (95%CI 1.27, 4.42)). VRE-infected patients had significantly longer mean ICU and total hospital stays (both *p* < 0.0001), with increased one-year mortality compared to cirrhotic patients without VRE infection, adjusted for age, sex, Hispanic ethnicity, and disease severity.

**Conclusions:**

It is unclear whether VRE infection serves as an independent risk factor for increased mortality or an indicator for patients with more severe illnesses and thus a higher risk for death.

## Background

Vancomycin resistant enterococci (VRE) have emerged as a worldwide concern especially in patients hospitalized in the Intensive Care Unit setting and in those who are immunocompromised [1]. Among liver transplant recipients, the most common bacterial pathogens include Enterococcus species and Enterobacteriaceae species [1]. As fluoroquinolones have increasingly been in use for prophylaxis in spontaneous bacterial peritonitis (SBP), there has been an increasing trend towards more gram-positive bacterial infections compared to gram-negative organisms [2]. Patients with cirrhosis who are awaiting liver transplant or have received liver transplants, are at an increased risk of colonization and infection with VRE [3]. Several studies involving liver transplant recipients have shown increased morbidity and mortality in patients with VRE infection and colonization compared to those without VRE [1, 3, 4].

Fewer studies have evaluated the impact of VRE colonization and infection in liver transplant candidates prior to transplantation. McNeil et al. found that liver transplant candidates colonized with VRE before transplantation had greater morbidity, significantly longer time in the ICU after transplantation and longer post-operative hospital stays, but not greater mortality compared with non-colonized candidates [5]. This study did not compare outcomes of VRE-infected patients with patients infected with other organisms. In addition to SBP prophylaxis, cirrhotic patients with recurrent hepatic encephalopathy are commonly prescribed rifaximin, a non-absorbable antibiotic that decreases the rate of relapse of encephalopathy [6]. The impact of SBP prophylactic antibiotic use on VRE infection in cirrhotic patients has not been evaluated.

A retrospective, chart review study was performed involving all cirrhotic patients admitted to the Hepatology Service at Keck Hospital of the University of Southern California from January 2010 through December 2014. The goals of this study were: [1] to examine the risk factors that predispose cirrhotic patients to VRE infection compared to cirrhotic patients infected with gram-negative organisms and uninfected patients; and [2] to use VRE infection status as an exposure variable to compare the subsequent outcomes, including 30-day and 1-year mortality, length of hospital stay, and length of ICU stay, of VRE-infected cirrhotic patients to other cirrhotic patients infected with gram-negative organisms and uninfected patients.

## Methods

### Setting and study population

The Keck Hospital of University of Southern California (USC) in Los Angeles, California is a 250-bed private, teaching hospital. All cirrhotic patients age 18 years or older, who were admitted to the Hepatology Service between January 2010 and December 2014 were analyzed. All aspects of this study received prior approval of the Institutional Review Board of the USC Health Sciences Campus.

### Objectives

The first objective was to use data collected at the time of determination of VRE infection to determine the risk factors associated with acquisition of VRE infection in cirrhotic patients. The second objective was to follow patients for hospital and mortality outcomes to evaluate if there is an increased risk of mortality at 30 days and at 1 year after VRE infection as well as length of hospital and ICU stay.

### Study design and data collection

This was a retrospective chart review study involving 533 consecutive patients with cirrhosis admitted to the Hepatology Service between January 2010 and December 2014. Demographics and laboratory data were extracted from electronic health records.

### VRE-infected patients and non VRE-infected comparators

Patients were categorized into three groups based on the results of cultures from blood, urine, ascites, pleural fluid, BAL and wounds. VRE cases, comparator group 1 (gram-negative infections) and comparator group 2 (no infections). VRE positivity was inclusive of both *Enterococcus faecalis* and *Enterococcus faecium* strains, identified using the Vitek..Variables recorded at the time of hospitalization and determination of infection status included demographics, history of antibiotic prophylaxis and presence of co-morbidities at 30 days prior (or closest to 30 days prior) to infection with VRE or negative culture. Comorbidities reviewed included diabetes, need for hemodialysis, presence of ascites, history of gastro-intestinal bleed or SBP. Laboratory parameters included creatinine, white blood cell (WBC) count, neutropenia < 900/mm^3^, and liver tests. The review of antibiotic history included use of prophylactic antibiotics for SBP and rifaximin or neomycin for hepatic encephalopathy. The severity of cirrhosis was determined by calculating the Model for End-Stage Liver Disease score (MELD) and Child Pugh score. The etiology of cirrhosis was also recorded. For VRE positive patients, the type of treatment received for VRE was noted (daptomycin, linezolid, synercid, tigecycline or no treatment). Whether subsequent organ transplant was performed after the positive or negative culture in question was determined. For the hospital admission during which time the positive or negative culture occurred, length of total hospitalization stay and length of ICU stay were recorded. Date of last follow-up and/or date of death were noted to ascertain death or survival at 30 days and at 1 year after the identified positive or negative culture.

### Statistical methods

Objective 1 (determine the risk factors associated with acquisition of VRE infection in cirrhotic patients). VRE positive patients were compared to two different l groups: VRE positive compared to sterile, and VRE positive compared to patients with gram-negative infection. Descriptive statistics involved presentations of group means (with standard deviations) for continuous variables and frequencies (with percentages) for categorical variables. Initial comparisons on descriptive statistics used independent t-tests and Fisher’s exact test to compare each VRE negative subset group to the VRE positive patients.

Associations of demographic and clinical variables with the presence/absence of VRE infection were estimated and tested using logistic regression; associations were summarized as odds ratios (OR) and 95% confidence intervals. Univariate associations with each of the independent variables were first evaluated. Continuous variables of age and WBC were categorized into 4-level variables based on the quartile distribution of each variable; odds ratios for the second through fourth quartile categories were estimated, estimating the odds of VRE infection relative to the first quartile. Creatinine (< 1.5, ≥1.5) and MELD score (≤20, 21–30, ≥31) were categorized by clinically relevant categories; odds ratios for the upper categories were estimated relative to the lowest category. The dialysis association was estimated for any dialysis relative to no dialysis. Multivariable models included all independent variables significant on univariate testing at *p* < 0.05. Other independent variables with univariate associations of *p* < 0.20 were alternately added into the model. The final multivariable model included all variables jointly significant at *p* < 0.05.

Objective 2 (evaluate VRE association with mortality and length of stay outcomes). VRE infection status was used as the primary independent exposure variable to evaluate mortality and length of stay outcomes. VRE positive and negative patients were compared on 1-year and 30-day mortality with logistic regression. Mortality analyses (as the dependent outcome) were limited to subjects who were followed for the defined follow-up period (i.e., follow-up of at least 30 days for 30-day mortality, and at least 1 year for 1-year mortality); results are reported as odds ratios (odds of mortality in VRE positive versus VRE negative patients) with 95% confidence interval. The average length of hospitalization was compared between VRE positive and negative groups using truncated negative binomial regression for count data (truncation to account for zero not being a possible value). The average length of ICU stay was compared by negative binomial regression for count data. Because VRE positive patients were more likely than VRE negative patients to be of Hispanic ethnicity and female and to have a Child Pugh score of C, all associations of VRE infection with mortality and length of stay outcomes were estimated with and without adjustment for Hispanic ethnicity, gender and Child Pugh score.

## Results

### Patient characteristics

A total of 533 patients consecutively admitted to the Hepatology Service with cirrhosis were identified from the period of January 2010 through December 2014. 65 patients were infected with VRE, 80 patients were infected with gram-negative organisms (comparator 1) and cultures were sterile in 306 patients (comparator 2) (Table 1) yielding an analysis sample of 451 patients. The additional 82 patients who were VRE negative, but had gram positive cultures were excluded from this analysis, as we did not feel they were clinically relevant. A higher proportion of women were VRE positive compared to patients with VRE negative cultures (Table 1). VRE positive patients were more likely to be Child class C and with higher MELD scores than those with gram-negative infections or sterile cultures. The majority of the patients who had positive VRE cultures had this organism isolated from the urine whereas equal numbers of patients infected with other organisms (comparator 1) had organisms isolated from urine and blood.

**Table 1:** VRE Sample Demographics (*n* = 533)

VRE Status			
Variable	VRE Positive (*n* = 65)	VRE Negative, Gram Negative (*n* = 80)	VRE Negative, Sterile (*n* = 306)
Age	53.4 (11.1)^a^	56.2 (10.6)	55.5 (9.7)
Sex			
Male	24 (36.9%)	43 (53.7%)	205 (67.0%)
Female	41 (63.1%)	37 (46.3%)	101 (33.0%)
Race^b^			
White	17 (26.1%)	22 (27.5%)	117 (38.2%)
African-American	1 (1.5%)	5 (6.3%)	6 (2.0%)
Hispanic	41 (63.1%)	47 (58.7%)	145 (47.4%)
Asian	4 (6.2%)	4 (5.0%)	23 (7.5%)
Other	2 (3.1%)	2 (2.5%)	15 (4.9%)
Child Pugh Score^b^			
A	1 (1.5%)	9 (11.2%)	27 (8.8%)
B	14 (21.5%)	28 (35.0%)	127 (41.5%)
C	50 (76.9%)	43 (53.8%)	152 (49.7%)
MELD Score^b^	25.4 (9.9)	20.3 (8.8)	19.7 (9.7)
Culture Site^b^			
Blood	12 (18.5%)	20 (43.5%)	62 (49.6%)
Urine	43 (66.1%)	20 (43.5%)	47 (37.6%)
Ascitic fluid	4 (6.2%)	4 (8.7%)	14 (11.2%)
Pleural fluid	0	0	0
BAL	5 (7.7%)	2 (4.3%)	1 (0.8%)
Wound	1 (1.5%)	0	1 (0.8%)
Transplant History^b^			
None	48 (73.8%)	62 (77.5%)	238 (78.6%)
Liver	13 (20.0%)	13 (16.3%)	57 (18.8%)
Liver/kidney	4 (6.2%)	5 (6.2%)	8 (2.6%)
VRE Treatment^b^			
No treatment	12 (19.1%)		
Linezolid	36 (57.1%)		
Daptomycin	11 (17.5%)		
Tigecycline	4 (6.3%)		

^a^Numbers in table are mean (SD) for age; *n* (%) for categorical variables

^b^VRE treatment missing in 2 VRE positive subjects; culture site missing in 34 g negative subjects and in 181 VRE negative, sterile subjects; Meld score missing in 1 VRE negative, sterile subject; transplant history missing in 3 VRE negative, sterile subjects

### Risk factors for VRE infection

To assess the risk factors associated with acquisition of VRE infection, logistic regression analyses were performed on VRE cases compared to the two VRE-negative comparator groups (Table 2). Female gender, severity of liver disease measured by higher Child Pugh and MELD scores, presence of ascites and SBP, use of any type of dialysis, SBP prophylaxis (fluoroquinolone and TMP/SMX) and rifaximin use were positively associated with VRE infection. The use of any antibiotic (either as prophylaxis for SBP or hepatic encephalopathy) was associated with an increased risk of VRE infection compared to uninfected patients (*p* = 0.003) (Table 2). Patients with Child Pugh class C were more likely to be infected with VRE compared to those with Child Pugh class A or B (*p* < 0.001). Neither age, diabetes, history of gastrointestinal bleed or etiologies of cirrhosis were significant risk factors for acquisition of VRE infection. Regarding laboratory parameters, neutropenia less than or equal to 900/mm^3^ and creatinine level were not correlated with VRE or gram-negative infections.

**Table 2:** Univariate (Unadjusted) Associations with VRE infection: VRE Positive Compared to VRE Negative, Gram Negative, and VRE Negative, Sterile

Variable	VRE Positive (*n* = 65)	VRE Negative, Gram Negative (*n* = 80)	OR (95% CI)	*p*-value	VRE Negative, Sterile (*n* = 306)	OR (95% CI)	*p*-value
Age							
< 47	17 (26.1%)	15 (18.8%)	1.0	0.56	52 (17.0%)	1.0	0.30
47–53	10 (15.4%)	12 (15.0%)	0.74 (0.25, 2.19)		61 (19.9%)	0.50 (0.21, 1.19)	
54–60	18 (27.7%)	20 (25.0%)	0.79 (0.31, 2.04)		78 (25.5%)	0.71 (0.33, 1.49)	
≥ 60	20 (30.8%)	33 (41.2%)	0.53 (0.22, 1.30)		115 (37.6%)	0.53 (0.26, 1.10)	
Sex							
Male	24 (36.9%)	43 (53.7%)	1.0		205 (67.0%)	1.0	
Female	41 (63.1%)	37 (46.3%)	1.99 (1.02, 3.87)	0.04	101 (33.0%)	3.47 (1.99, 6.05)	< 0.001
Hispanic							
No	24 (36.9%)	33 (41.2%)	1.0		161 (52.6%)	1.0	
Yes	41 (63.1%)	47 (58.8%)	1.20 (0.61, 2.35)	0.60	145 (47.4%)	1.90 (1.09, 3.29)	0.02
Albumin							
< 2.5	12 (18.5%)	21 (26.9%)	1.0		67 (22.0%)	1.0	
2.5 or higher	53 (81.5%)	57 (73.1%)	1.63 (0.73, 3.63)	0.23	237 (78.0%)	1.25 (0.63, 2.47)	0.52
Neutropenia^a^							
< 900	1 (1.5%)	1 (1.2%)	1.0		1 (0.3%)	1.0	
900 or higher	64 (98.5%)	79 (98.8%)	0.81 (0.05, 13.21)	0.32	303 (99.7%)	0.21 (0.01, 3.42)	0.32
Child Pugh Score							
A	1 (1.5%)	9 (11.3%)	1.0	0.004	27 (8.8%)	1.0	< 0.001
B	14 (21.5%)	28 (35.0%)	4.50 (0.52, 39.15)		127 (41.5%)	2.98 (0.38, 23.61)	
C	50 (76.9%)	43 (53.7%0	10.47 (1.27, 85.96)		152 (49.7%)	8.88 (1.18, 67.04)	
Child Pugh Score (dichotomized)							
A or B	15 (23.1%)	37 (46.2%)	0.35 (0.17, 0.72)	0.003	154 (50.3%)	0.30 (0.16, 0.55)	< 0.001
C	50 (76.9%)	43 (53.8%)	1.0		152 (49.7%)	1.0	
MELD Score^b^							
≤ 20	24 (36.9%)	46 (57.5%)	1.0	0.036	182 (59.9%)	1.0	0.0015
21–30	22 (33.9%)	21 (26.2%)	2.01 (0.92, 4.36)		22 (27.2%)	2.11 (1.12, 3.99)	
≥ 31	19 (29.2%)	13 (16.3%)	2.80 (1.18, 6.63)		13 (16.0%)	3.35 (1.68, 6.66)	
Diabetes Mellitus							
No	46 (70.8%)	51 (63.8%)	1.0		212 (69.3%)	1.0	
Yes	19 (29.2%)	29 (36.2%)	0.73 (0.36, 1.47)	0.37	94 (30.7%)	0.93 (0.52, 1.68)	0.81
Ascites							
No	5 (7.7%)	33 (41.3%)	1.0		91 (29.7%)	1.0	
Yes	60 (92.3%)	47 (58.7%)	8.43 (3.05, 23.25)	< 0.001	215 (70.3%)	5.08 (1.97, 13.06)	< 0.001
History of GI Bleed							
No	45 (69.2%)	58 (70.0%)	1.0		223 (72.9%)	1.0	
Yes	20 (30.8%)	24 (30.0%)	1.04 (0.51, 2.11)	0.92	83 (27.1%)	1.19 (0.67, 2.14)	0.55
History of SBP							
No	53 (81.5%)	70 (87.5%)	1.0		283 (92.8%)	1.0	
Yes	12 (18.5%)	10 (12.5%)	1.58 (0.64, 3.95)	0.32	22 (7.2%)	2.91 (1.36, 6.24)	0.009
Dialysis							
None	48 (73.9%)	69 (86.2%)	1.0	0.007	268 (87.6%)	1.0	< 0.001
PD	0 (0%)	0 (0%)	–		1 (0.3%)	–	
HD	8 (12.3%)	10 (12.5%)	1.15 (0.42, 3.13)		35 (11.4%)	1.28 (0.56, 2.92)	
CRRT	9 (13.8%)	1 (1.3%)	12.94 (1.59, 105.50)		2 (0.7%)	25.12 (5.27, 119.88)	
Any Dialysis							
No	48 (73.8%)	69 (86.3%)	1.0		268 (87.6%)	1.0	
Yes	17 (26.2%)	11 (13.7%0	2.22 (0.96, 5.16)	0.06	38 (12.4%)	2.50 (1.31, 4.78)	0.008
WBC							
< 4.2	10 (15.4%)	24 (30.0%)	1.0	0.09	81 (26.6%)	1.0	0.06
4.2–5.7	12 (18.5%)	19 (23.7%)	1.52 (0.54, 4.26)		77 (25.2%)	1.26 (0.52, 3.09)	
5.8–8.0	21 (32.3%)	19 (23.8%)	2.65 (1.01, 6.96)		65 (21.3%)	2.62 (1.15, 5.95)	
≥ 8.1	22 (33.8%)	18 (22.5%)	2.93 (1.12, 7.70)		82 (26.9%)	2.17 (0.97, 4.88)	
Creatinine^b^							
< 1.5	38 (58.5%)	58 (72.5%)	1.0		208 (68.2%)	1.0	
≥ 1.5	27 (41.5%)	22 (27.5%)	1.87 (0.93, 3.76)	0.08	97 (31.8%)	1.52 (0.88, 2.64)	0.14
SBP Prophylaxis							
None	44 (67.7%)	65 (81.2%)	1.0	0.13	253 (82.7%)	1.0	0.034
TMP/SMX	6 (9.2%)	7 (8.8%)	1.27 (0.40, 4.02)		22 (7.2%)	1.57 (0.60, 4.09)	
Fluoroquinolone	14 (21.5%)	8 (10.0%)	2.59 (1.00, 6.68)		31 (10.1%)	2.60 (1.28, 5.27)	
Other	1 (1.5%)	0	–		0	–	
Any SBP Prophylaxis^c^							
No	44 (67.7%)	65 (81.2%)	1.0		253 (82.7%)	1.0	
Yes	21 (32.3%)	15 (18.8%)	2.07 (0.96, 4.45)	0.06	53 (17.3%)	2.28 (1.25, 4.14)	0.009
Rifaximin							
No	39 (60.0%)	55 (68.7%)	1.0		229 (74.8%)	1.0	
Yes	26 (40.0%)	25 (31.3%)	1.47 (0.74, 2.91)	0.27	77 (25.2%)	1.98 (1.13, 3.47)	0.02
Neomycin							
No	61 (93.8%)	73 (92.4%)	1.0		294 (96.1%)	1.0	
Yes	4 (6.2%)	6 (7.6%)	0.80 (0.22, 2.96)	0.73	12 (3.9%)	1.61 (0.50, 5.15)	0.44
Any Antibiotic^d^							
No	27 (41.5%)	40 (50.0%)	1.0		188 (61.4%)	1.0	
Yes	38 (58.5%)	40 (50.0%)	1.41 (0.73, 2.72)	0.31	118 (38.6%)	2.24 (1.30, 3.86)	0.003
Etiology							
ETOH	19 (29.2%)	26 (32.5%)	1.0	0.90	106 (34.6%)	1.0	0.21
Hep B/C	23 (35.4%)	28 (35.0%)	1.12 (0.50, 2.52)		125 (40.9%)	1.03 (0.53, 1.99)	
Other	23 (35.4%)	26 (32.5%)	1.21 (0.54, 2.74)		75 (24.5%)	1.71 (0.87, 3.36)	

^a^Neutropenia comparison to VRE negative, gram negative: *p*-value by exact logistic regression

^b^Missing in 1 VRE negative, sterile subject

^c^Includes TMP/SMX, fluoroquinolone, other prophylactic treatment

^d^Includes SBP prophylaxis, rifaximin, neomycin

Multivariate analysis revealed that significant risk factors for acquisition relative to the VRE negative, gram-negative comparison group included female sex, higher Child Pugh, presence of ascites, and dialysis (Table 3).

**Table 3:** Multivariable Associations with VRE infection

Variable	OR (95% CI)	*p*-value
a. VRE Infected vs. VRE Negative, Gram Positive		
Female	3.73 (1.64, 8.49)	0.002
Child Pugh A or B (vs C)	0.37 (0.16, 0.84)	0.018
Ascites	9.43 (3.22, 27.65)	< 0.001
Dialysis	3.31 (1.21, 9.04)	0.02
b. VRE Infected vs. VRE Negative, Sterile		
Age		
< 47	1.0	0.041
47–53	0.32 (0.12, 0.88)	
54–60	0.57 (0.24, 1.36)	
≥ 60	0.32 (0.14, 0.75)	
Female	5.60 (2.90, 10.80)	< 0.001
Child Pugh A or B (vs C)	0.42 (0.21, 0.83)	0.013
Ascites	4.86 (1.74, 13.61)	0.003
Dialysis	3.19 (1.48, 6.87)	0.003
Any Antibiotic	2.37 (1.27, 4.42)	0.007

### VRE infections and outcomes

After adjusting for age, ethnicity, gender, MELD and Child Pugh scores, VRE infection was not associated with statistically significant higher 30-day mortality (age-, gender-, and ethnicity-adjusted OR [95% CI] = 1.66 [0.61, 4.50] compared to other gram-negative, and 1.27 [0.57, 2.85] compared to uninfected individuals) (Table 4); adjustment for disease severity further reduced the associations. Adjusted for age, ethnicity, and sex, 1-year mortality was significantly higher in the VRE positive group (adjusted OR = 2.96 [1.23, 7.14] compared to other gram-negative (*p* = 0.02), and 2.87 [1.39, 5.93) compared to uninfected individuals (*p* = 0.005)). Further adjustment for disease severity reduced these associations to elevated but statistically non-significant levels (for gram-negative comparator, adjusted OR = 2.06 [0.81, 5.27]; for uninfected comparator, adjusted OR = 2.04 [0.95, 4.38]). Nonetheless, VRE infection resulted in a significantly longer length of stay in the ICU and overall hospitalization compared to patients infected with other organisms (*p* < 0.001) or uninfected patients (*p* < 0.001) (Table 4). These differences remained significant after adjusting for age, ethnicity, gender, and disease severity scores.

**Table 4:** Comparison of Morbidity and Mortality by VRE infection

Outcome^a^	VRE Positive	VRE Negative, Gram Negative	*p*-value	VRE Positive	VRE Negative, Sterile	*p*-value
1-year mortality^b^						
Mortality, *n* (%)	22 (48.9%)	18 (30.5%)		22 (48.9%)	44 (26.7%)	
Unadjusted OR (95% CI)	2.18 (0.97, 4.88)		0.06	2.63 (1.33, 5.19)		0.005
OR (95% CI) adjusted for:						
Age, Hispanic, Sex	2.96 (1.23, 7.14)		0.02	2.87 (1.39, 5.93)		0.005
Age, Hispanic, Sex, Child Pugh	2.06 (0.81, 5.27)		0.13	2.04 (0.95, 4.38)		0.07
Age, Hispanic, Sex, Meld	2.26 (0.88, 5.84)		0.09	1.97 (0.88, 4.41)		0.10
30-day mortality^c^						
Mortality, *n* (%)	10 (17.9%)	10 (13.5%)		10 (17.9%)	36 (14.9%)	
Unadjusted OR (95% CI)	1.39 (0.54, 3.62)		0.50	1.24 (0.57, 2.67)		0.59
OR (95% CI) adjusted for						
Age, Hispanic, Sex	1.66 (0.61, 4.50)		0.32	1.27 (0.57, 2.85)		0.56
Age, Hispanic, Sex, Child Pugh	1.10 (0.39, 3.15)		0.86	0.74 (0.32, 1.72)		0.49
Age, Hispanic, Sex, Meld	1.09 (0.36, 3.30)		0.88	0.58 (0.23, 1.46)		0.25
Hospital length of stay (days)						
Unadjusted mean days (95% CI)	24.8 (18.4, 31.3)	10.7 (8.1, 13.3)	< 0.001	25.4 (19.6, 31.2)	7.7 (6.8, 8.6)	< 0.001
Mean days (95% CI) adjusted for						
Age, Hispanic, Sex	25.3 (18.4, 32.3)	10.6 (7.9, 13.2)	< 0.001	24.3 (18.6, 30.0)	7.8 (6.9, 8.7)	< 0.001
Age, Hispanic, Sex, Child Pugh	25.1 (18.4, 31.8)	10.7 (8.1, 13.4)	< 0.001	23.8 (18.3, 29.3)	7.9 (7.0, 8.8)	< 0.001
Age, Hispanic, Sex, Meld	24.7 (18.2, 31.2)	11.2 (8.3, 14.0)	< 0.001	21.9 (17.0, 26.8)	8.2 (7.2, 9.2)	< 0.001
ICU length of stay (days)						
Unadjusted mean days (95% CI)	12.9 (6.0, 19.8)	3.3 (1.7, 4.9)	< 0.001	12.9 (5.9, 19.9)	2.5 (1.8, 3.1)	< 0.001
Mean days (95% CI) adjusted for						
Age, Hispanic, Sex	14.3 (6.0, 22.5)	3.1 (1.6, 4.6)	< 0.001	12.6 (5.7, 19.6)	2.5 (1.3, 3.1)	< 0.001
Age, Hispanic, Sex, Child Pugh	15.8 (6.4, 25.1)	3.0 (1.5, 4.5)	< 0.001	12.3 (5.5, 19.1)	2.6 (1.9, 3.3)	< 0.001
Age, Hispanic, Sex, Meld	14.0 (5.5, 22.6)	3.1 (1.4, 4.8)	< 0.001	10.4 (4.8, 15.9)	2.7 (1.8, 3.5)	< 0.001

^a^1-year and 30-day mortality compared by logistic regression; hospital length of stay compared by zero-truncated negative binomial regression; ICU length of study compared by negative binomial regression. Meld covariate 3-level (≤20, 21–30, ≥31).

^b^1-year mortality evaluated among 104 (45 VRE-positive, 59 VRE- gram negative) subjects with minimum of 1-year follow-up. 1-year mortality evaluated among 210 (45 VRE-positive, 165 VRE-negative, sterile) subjects with minimum of 1-year follow-up.

^c^30-day mortality evaluated among 130 (56 VRE-positive, 74 VRE- gram negative) subjects with minimum of 30-day follow-up. 30-day mortality evaluated among 297 (56 VRE-positive, 241 VRE-negative, sterile) subjects with minimum of 30-day follow-up.

## Discussion

VRE infection is a significant cause of morbidity in patients with end stage liver disease [1]. The continued spread of multi-drug resistant bacteria leads to limited treatment options and potential for significant negative impact on patients. It has been well documented that VRE colonization and infection in orthotopic liver transplant (OLT) recipients results in increased morbidity, as defined by longer ICU stay, and mortality [3, 5, 7–9]. Studies have shown that VRE colonization in cirrhotics is associated with increased risk of subsequent infection and death [5, 10]. However, there is a deficit of literature addressing the outcomes of cirrhotic patients prior to transplantation who are infected with VRE and the risk factors associated with acquisition of VRE infection.

In this ethnically diverse retrospective study, VRE infected and non-infected populations significantly differed with respect to gender, race and disease severity indices. The majority of the VRE infected subjects were female, Hispanic race and had higher Child Pugh and MELD scores compared to those who were infected with other organisms (non-VRE) or had sterile cultures.

Female gender and Hispanic race have not previously been shown to be risk factors for VRE acquisition in cirrhotic patients. The reasoning for why women and Hispanics are at increased risk was not evidently clear. It is possible that the occurrence is due in part to a high Hispanic population in southern California leading to an overall increased prevalence of the disease. As many VRE cultures were collected from urine, one could predict a female predominance given incidence of urinary tract infection being more common in females [11].

In the univariate analysis compared to uninfected individuals, the use of SBP prophylaxis was found to be a risk factor for the acquisition of VRE infection as was the use of rifaximin, a non-absorbable antibiotic used to treat hepatic encephalopathy [6]. When all antibiotics were evaluated together, the use of any antibiotic (either for SBP or hepatic encephalopathy prophylaxis) was found to be a risk factor for VRE infection, compared to uninfected individuals. VRE colonization is associated with antibiotic usage, in particular prolonged fluoroquinolone use in cirrhotic patients, and has been associated with increased risk of gram-positive infections [2, 8]. Hagen et al. evaluated the role that pre-transplant antibiotics, in particular SBP prophylaxis, had with VRE colonization rates [12]. Cirrhotic patients with moderate illness (median MELD 11) had VRE rectal swab cultures and completed screening questionnaires to determine antibiotic exposure during the 3 months prior to study enrollment. Only 3.4% of the participants were colonized with VRE, and 17% had taken SBP prophylaxis (specific antibiotics were not mentioned) during the prior 3 months, showing no correlation of SBP prophylaxis on VRE colonization. Not all participants had cultures collected at other sites to indicate actual VRE infection, and of the few reported, most were actually not colonized with VRE. As such, the authors suggest that pre-transplant colonization with VRE plays a minor role in post-transplant morbidity and mortality attributed to the VRE infection [12]. Thus, it appears that measures aimed at reducing VRE colonization should be directed to more critically ill patients with higher MELD scores. This is not a practice we do at our hospital to routinely check rectal swabs. Another study found the use of SBP prophylaxis with fluoroquinolones in cirrhotics was more predictive of infection with antibiotic resistant bacterial organism, notably VRE [13]. Interestingly, this same study did not find a significant risk of antibiotic resistant bacterial infection with the use of non-absorbed antibiotics such as rifaximin. Another study showed that the use of the non-absorbed antibiotic, neomycin, was associated with VRE colonization [5]. These findings are in contrast with our results, as ours showed that rifaximin use was associated with increased risk of infection with VRE. This is an interesting result given the characteristics of rifaximin as having an estimated bioavailability in the blood after oral administration of less than 0.4%. When in anaerobic environment such as the colon, rifaximin reduces plasmid transfer, which is important in transfer of bacterial resistance [7]. With guidelines now recommending rifaximin as the first line treatment for hepatic encephalopathy, this raises concern for potential increases in development of bacterial resistance in a time when rifaximin use will only continue to intensify [6].

Lastly, our analysis showed that any dialysis (HD, PD or CRRT) was associated with increased risk of VRE infection (Table 3) compared both the gram negative and uninfected individuals. While dialysis may be considered a surrogate for the overall severity of illness among those patients with VRE infection, the strong positive association of VRE infection with dialysis remained after adjustment for disease severity measured as the Child Pugh score in multivariate analysis (Table 3).

All of the patients were followed for 1 year after the identified positive or negative culture to document subsequent transplantation. It was interesting to note that the majority of all patients in the study actually did not go on to receive any type of transplantation at our study center.

Multiple studies have shown that VRE infection in liver transplant recipients results in longer hospital stay and mortality [3, 5, 7–9]. Similarly, we show that VRE infection in cirrhotics pre-transplantation have longer mean ICU and mean total hospital stay in addition to increased one-year mortality compared to those cirrhotics without VRE infection (Table 4). This was true even after adjustment for sex, race and ethnicity, but was reduced to non-significant levels with adjustment for severity indices (Child Pugh and MELD scores). It is unclear whether VRE infection serves as an independent risk factor for increased mortality or if it is an indicator for patients with more severe illnesses and thus at a higher risk for death. Nonetheless, identification of VRE should prompt timely therapy and implementation of appropriate preventive strategies.

Limitations to our study include the retrospective study data acquisition comprised of chart review, and sampling from a single referral center. Furthermore, some follow up may be incomplete in a few subjects in regards to mortality, as continued care is not always done at our facility if patients do not go on to transplant. Our data may be incomplete in regards to specific details of the medical history and prior antibiotics obtained from outside hospitals, as our institution is a referral center so not all records were available or accessible for the patient during the chart review. While this study employed a small patient sample with subsequent reduction in statistical power, it is still the largest series of cirrhotics analyzed with VRE. This is one of the first studies that showed the effect of female gender and Hispanic ethnicity on VRE. There will need to be further studies completed in order to further elucidate the role of cirrhosis and VRE on outcomes particularly after liver transplantation.

## Conclusions

Hispanic ethnicity and female gender in this study was associated with VRE infection in cirrhotic patients admitted to Keck Medical Center of the University of Southern California. Due to the morbidity associated with VRE infection it is important to be aware of these risk factors and further studies into these infections.

## Data Availability

The datasets used and/or analyzed during the current study are available from the corresponding author on reasonable request.

## Abbreviations

ICU: Intensive Care Unit
MELD: Model for End-Stage Liver Disease
OR: Odds Ratio
SBP: Spontaneous Bacterial Peritonitis
USC: University of Southern California
VRE: Vancomycin-resistant enterococcus
WBC: White blood cell
